# Is Plasma Total Antioxidant Capacity Elevated in Professional Soccer Athletes?: A Cross-Sectional Study

**DOI:** 10.3390/sports14020045

**Published:** 2026-01-23

**Authors:** Tomoharu Mochizuki, Takashi Ushiki, Hikaru Kanome, Takumu Tsuchida, Mami Osawa, Misato Sato, Hajime Ishiguro, Tatsuya Suwabe, Satoshi Watanabe, Go Omori, Noriaki Yamamoto, Tomoyuki Kawase

**Affiliations:** 1 Department of Orthopaedic Surgery, Graduate School of Medical and Dental Sciences, Niigata University, Niigata 951-8510, Japan; tommochi121710@gmail.com; 2 Division of Hematology and Oncology, Graduate School of Health Sciences, Niigata University, Niigata 951-8518, Japan; tushiki@med.niigata-u.ac.jp (T.U.); hikakanome@gmail.com (H.K.); oswmami@clg.niigata-u.ac.jp (M.O.); 3 Department of Transfusion Medicine, Cell Therapy and Regenerative Medicine, Niigata University Medical and Dental Hospital, Niigata 951-8520, Japan; misatosato.zd8@nuh.niigata-u.ac.jp; 4 Department of Hematology, Endocrinology and Metabolism, Faculty of Medicine, Niigata University, Niigata 951-8510, Japan; takumukota@yahoo.co.jp (T.T.); power@med.niigata-u.ac.jp (H.I.); tsuwabe@med.niigata-u.ac.jp (T.S.); 5 Kobarizaka Clinic, Niigata 950-2011, Japan; wwsatoshiww@gmail.com; 6 Department of Health and Sports, Faculty of Health Sciences, Niigata University of Health and Welfare, Niigata 950-3198, Japan; omori@nuhw.ac.jp; 7 Niigata Rehabilitation Hospital, Niigata 950-3304, Japan; nirehp.yamamoto@aiko.or.jp; 8 Division of Oral Bioengineering, Institute of Medicine and Dentistry, Niigata University, Niigata 951-8514, Japan

**Keywords:** professional athletes, energetics, total antioxidant capacity, body composition indexes, anemia, thrombocytopenia, platelet-rich plasma

## Abstract

Owing to its ability to promote early recovery, platelet-rich plasma (PRP) is a popular regenerative therapy for treating sports injuries. However, its scientific basis has not yet been fully established. To bridge this gap, we focused on systemic antioxidant capacity, which suppresses exacerbated inflammation. Plasma total antioxidant capacity (TAC) in male professional soccer athletes (*n* = 30) was assessed alongside body composition measurements and compared with that of non-athletes (*n* = 31). Metabolic and inflammatory conditions were examined using erythrocyte sedimentation rate and plasma lactate, glucose, and protein levels. TAC levels were similar in both groups. Moderate correlations were observed between TAC and body fat percentage, skeletal muscle percentage, and basal metabolic rate in the control group, but not in the pro-athlete group, which exhibited superior body composition indices. These findings suggest that TAC levels may not directly influence PRP therapy in athletes. However, when compared with TAC levels calculated using correlations obtained in controls, the measured TAC levels (329.3 mM) were substantially higher than the compensated levels (62.5 mM for basal metabolic rate) in pro-athletes. This analytical approach indicates that TAC may theoretically be elevated to higher levels in pro-athletes when evaluated using non-athlete-based scales.

## 1. Introduction

Platelet-rich plasma (PRP) is increasingly used to treat patients with musculoskeletal disorders [1,2]. Despite often yielding conflicting outcomes, PRP is widely used to treat athletes at all levels, from recreational to Olympic [3,4,5], owing to its substantial benefits. PRP is expected to promote tissue regeneration and reduce inflammation and pain, thereby facilitating a rapid return to sports and decreasing the risk of re-injury [6,7]. Although not well documented in the scientific literature, many sports doctors have shared their clinical experience that PRP accelerates healing in athletes more than in sedentary adults of the same age group. To convince skeptical medical professionals and disseminate this therapeutic option, further studies are required to obtain strong evidence [8].

However, it is also true that PRP therapy has often yielded conflicting outcomes. Before discussing the possible reasons, the mode of PRP action should be reviewed. PRP treatment is a biological therapy based on the concept of platelet aggregation at injury sites, which stimulates wound healing by delivering growth factors [9,10,11]. This phenomenon is mimicked by PRP therapy, except in patients with severe systemic disorders, such as hemophilia, or those receiving specific medications, such as antithrombotic drugs related to platelets and coagulation. Although this wound-healing scenario appears to be universal across many mammalian species, why do non-responders to PRP therapy often appear? Preparation skills (i.e., PRP quality) and administrative protocols undoubtedly influence clinical outcomes. However, we have long focused on patients’ physical conditions rather than PRP quality. Even if PRP is optimally prepared, it may be less effective in patients (i.e., recipients) with unsuitable local factors, including platelet-related or systemic medical conditions, and vice versa. In previous studies [12,13,14,15], we observed distinct platelet energetics in pro-athletes, characterized by low growth factor levels and high anti-inflammatory cytokine levels in PRP samples from professional and semiprofessional athletes. However, each data point has a limited impact on the current understanding of PRP therapy, and the sum of these data points is insufficient to account for the success of PRP therapy in athletes. Furthermore, to date, similar studies with the same perspective have rarely been published. Thus, although many sports doctors using PRP therapy have been sharing similar clinical questions, our objective was not entirely justified. However, based on a review article [16], it is plausible that the athlete’s body has a more potent defense mechanism against inflammation that helps maintain lower inflammatory levels, an effect that may benefit tissue regeneration in pro-athletes.

Another factor influencing tissue regeneration may be age. Biological aging reduces stem cell quality and quantity, thereby decreasing tissue regeneration capacity [17,18,19]. Exercise and diet can effectively improve biological age in older humans [20,21,22,23,24]. Therefore, even within the same young generation, pro-athletes are expected to exhibit higher plasma total antioxidant capacity (TAC), sufficient to scavenge reactive oxygen species (ROS) overproduced during intense to moderate exercise, thereby maintaining oxidative balance [13] and preventing local and systemic inflammation [25,26]. However, when exercise is highly intensive or prolonged, it can cause muscle damage, fatigue, and reduced performance, depending on the individual’s capacity for ROS defense mechanisms [27,28,29]. On the other hand, to our knowledge, direct evidence of TAC’s involvement in PRP therapy has not been provided by clinical studies. However, several, but not many, preclinical animal or in vitro studies suggest this possibility [30,31]. We also confirmed the appreciable antioxidant capacity of PPP in an in vitro study [32]. Thus, based on these reports, we hypothesized that plasma TAC levels may be elevated in pro-athletes. To determine whether pro-athletes maintain higher TAC levels, we measured TAC in samples from professional soccer players, analyzed the data alongside body composition indices, compared them with those of non-athletes of the same generation, and adjusted pro-athletes’ TAC data (i.e., compensated TAC data) using the linear regressions obtained in the non-athletic controls.

## 2. Materials and Methods

### 2.1. Experimental Design

The study design and consent forms for all procedures (project identification code: 2021-0126) were approved by the Ethics Committee for Human Participants of Niigata University (Niigata, Japan) and complied with the Declaration of Helsinki of 1964, as revised in 2013. Informed consent was obtained from all participants prior to their participation in the study.

**Figure 1:**
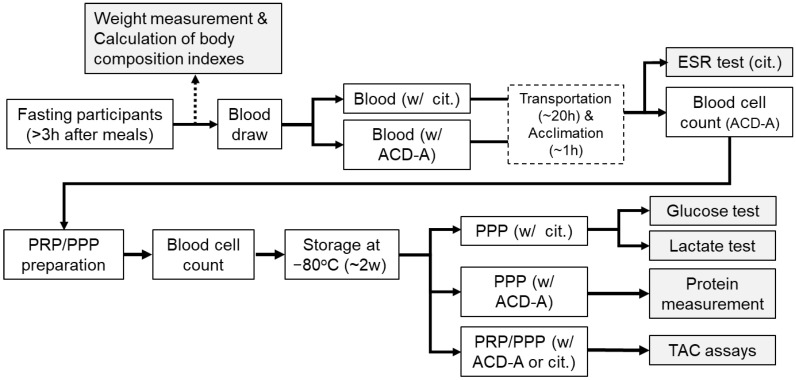
Experimental design and schedule.

This is a cross-sectional study between two groups. The experimental design and timetable, from blood collection to assays, are shown in Figure 1.

### 2.2. Study Variables

The variables examined in this study are listed below and briefly described.
Age:Chronological ageVisceral fat level (VFL): Classification of levels of visceral fatBody mass index (BMI):Measure of body fat based on height and weight Body fat percentage (BFP):Proportion of fat mass to total body massSkeletal muscle percentage (SMP):Proportion of skeletal muscle mass to total body massBone mass weight (BMW):Predicted weight of bone mineralBasal metabolic rate (BMR):Minimum energy (calories) the body burns at rest to perform essential life-sustaining functions like breathing, circulation, cell production, and temperature regulationMetabolic equivalent of task (MET): Unit measuring energy expenditure, representing how much more energy a physical activity uses compared to restingTotal antioxidant capacity (TAC):Overall ability to neutralize free radicals, indicating antioxidant defense power against oxidative stressMean platelet volume (MPV):Average size of your platelets, roughly indicating platelet age.Erythrocyte sedimentation rate (ESR):Speed of red blood cells (erythrocytes) settling in a tube, indicating inflammation in the body.

### 2.3. Participants in This Study

Almost all professional soccer players who joined a J1-class team, Albirex Niigata (2024−2025), in the Japan Professional Football League participated in this study. Age- and sex-matched non-athletic adults were enrolled based on inclusion and exclusion criteria. However, the candidates were limited to hospital workers and university students for convenience. Thus, participants could not be randomly selected from a large population.

Among participant candidates, healthy, non-smoking males were included in the study. As inclusion criteria, individuals positive for human immunodeficiency, hepatitis B, hepatitis C virus infection, or syphilis were excluded from the study. As exclusion criteria, individuals receiving medications, especially anticoagulants, antiplatelet drugs, and/or antioxidant-rich supplements or diets, with high oxidative stress levels, were also excluded from the study. These criteria were applied to both groups. Written informed consent was obtained from all participants. Because diet, including beverages and supplements, influences TAC [33], participants were required to limit dietary intake to 3 h before blood collection. In addition, participants who were not legally considered minors (18-year-olds) were excluded.

As shown in previous studies [13,15], despite frequent trading among players during and after the regular season, owing to regular intensive training and games, pro-athletes exhibited similar physical characteristics, such as skeletal muscle percentage (SMP) and basal metabolic rate (BMR).

### 2.4. Blood Collection

**Figure 2:**
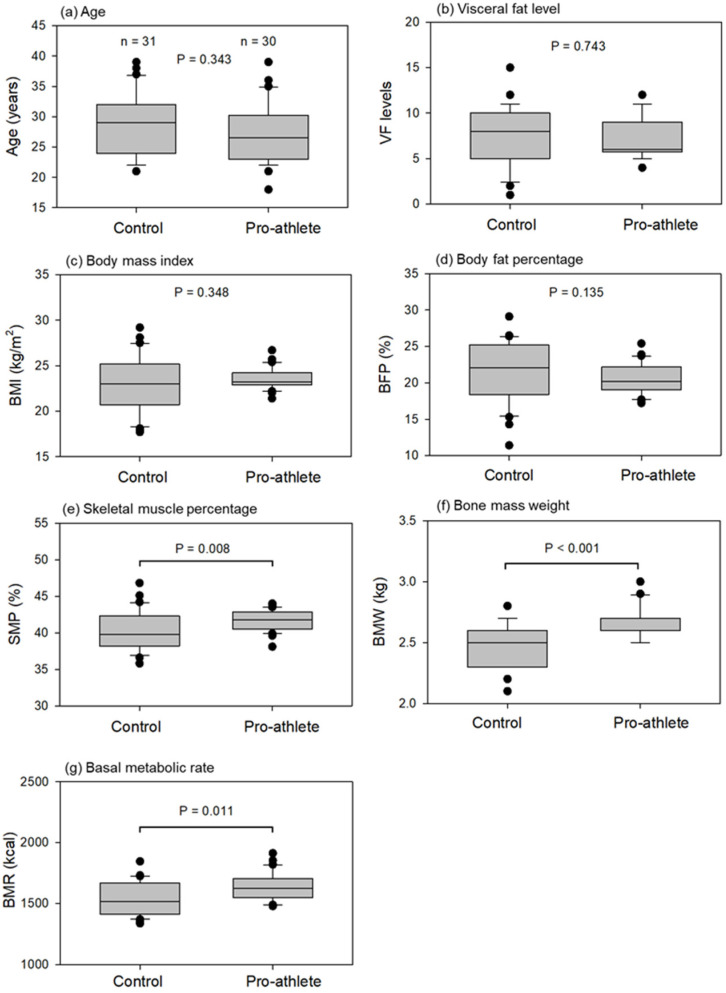
Age and body composition indices in the control and pro-athlete groups. (**a**) Age. (**b**) Visceral fat level (VFL). (**c**) Body mass index (BMI). (**d**) Body fat percentage (BFP). (**e**) Skeletal muscle percentage (SMP). (**f**) Bone mass weight (BMW). (**g**) Basal metabolic rate (BMR). Statistical analyses were conducted using the non-parametric Mann–Whitney U test. *n* = 31 (controls) or 30 (pro-athletes).

Blood samples were collected from male professional soccer athletes (*n* = 30) and sedentary adults of the same generation (*n* = 31) as controls in vacuum blood collection tubes containing the acid citrate dextrose A formulation (ACD-A; Vacutainer; Beckton, Dickinson and Company, Franklin Lakes, NJ, USA) or 3.8% sodium citrate (Venoject II; Terumo, Tokyo, Japan). The average age and other physical data are summarized in Figure 2. After the conclusion of the regular season, professional soccer players at resting state provided samples for medical examination.

Blood cell count, hemoglobin (HGB) level, hematocrit (HCT) value, and mean platelet volume were determined using an automated hematology analyzer (pocHi V-diff; Sysmex Corporation, Kobe, Japan). If a smooth curve was not observed on the platelet distribution histogram, the samples were discarded and not subjected to further experiments.

### 2.5. Preparation of Pure-PRP and PPP

Blood samples were transported to the laboratory for testing, and pure PRP and PPP samples were prepared within approximately 20 h. The samples were centrifuged horizontally at 415× *g* for 10 min (soft spin; Kubota, Tokyo, Japan). Upper plasma fractions, whose lower border was fixed at approximately 2 mm beyond the interface of the plasma and red blood cell (RBC) fractions, were collected as PRP samples. PRP samples were further centrifuged at 664× *g* for 4 min (hard spin) using an angle-type centrifuge (Sigma Laborzentrifugen, Osterode am Harz, Germany), and the resulting supernatants were collected as PPP samples. Both PRP and PPP samples were stored at −80 °C until use.

### 2.6. Determination of Plasma Glucose and Lactate Levels

Plasma lactate levels in PPP samples prepared from citrated blood were determined in duplicate with a lactate meter and specific test strips via the lactate oxidase enzyme electrode method (Lactate Pro2; Arkray, Kyoto, Japan).

Plasma glucose levels in PPP samples prepared using citrated blood samples were determined in duplicate using a glucose meter (FreeStyle Freedom Lite; NIPRO, Osaka, Japan).

### 2.7. Determination of Body Composition

Before blood collection, the participant’s body composition was determined using a bathroom weighing scale (HCS-FS03; ECLEAR, ELECOM, Osaka, Japan), as described previously [13,14,15]. This scale was installed with a unique magnetic resonance imaging-based program that accurately evaluated BFP based on the measured bioelectrical impedance and body weight. Using the manufacturer’s vast database, BMI, BFP, SMP, BMW, and BMR were automatically determined from the individual’s electrical impedance, body weight, and body height data.

### 2.8. Determination of Plasma TAC

PRP or PPP samples (20 μL) were thoroughly mixed with 180 μL of reaction buffer in a 96-well plate (TPP, Trasadingen, Switzerland), and initial absorbance was measured at 490 nm using a microplate reader (Model 680; Bio-Rad, Hercules, CA, USA). To initiate the reaction, 50 µL of copper ion reagent (OxiSelect TAC assay kit; Cell Biolabs, Inc., San Diego, CA, USA) was added to each well, and the plates were incubated on a shaker at room temperature (20–22 °C) for 5 min. The reaction was terminated by adding 50 µL of stop solution, and absorbance was measured again at 490 nm. Measurements were done in duplicate.

A standard curve was plotted using uric acid. TAC values of individual plasma samples were expressed as equimolar amounts of uric acid.

The raw data from pro-athletes were compensated based on the correlation coefficients obtained in controls (see the Section 3 for the regression equations). Briefly, each raw TAC value was substituted into each linear regression formula calculated from the correlation plot (SMP or BMR) to yield the compensated TAC value. It should be noted that this procedure was our tentative proposal and is not yet fully justified for data interpretation.

### 2.9. Determination of ESR

ESR was determined using the Westergren method. Briefly, whole blood samples were placed in vertically mounted tubes, and the length of RBC sedimentation was measured.

### 2.10. Quantification of Plasma Protein Levels

Plasma protein levels were determined in the citrated blood samples. PPP preparations were diluted 200-, 400-, and 600-fold with phosphate-buffered saline (PBS), mixed with Coomassie Brilliant Blue G-250 (Nacalai Tesque, Kyoto, Japan) in a 96-well plate, and subjected to the micro-Bradford assay. Absorbance was measured in duplicate at 595 nm using a microplate reader (Bio-Rad).

A standard curve was plotted using bovine serum albumin Fraction V (Sigma, St. Louis, MO, USA). Absorbance in the linear range was adopted for each representative plasma protein level.

### 2.11. MET Score

Individual exercise volumes were estimated using self-reported MET values in the questionnaire. The exercise type was quantified based on the compendium of physical activities proposed by Ainsworth et al. [34]. For example, in the sports category, a MET score of 10.0 is provided for “competitive soccer.” The US Centers for Disease Control and Prevention (CDC) defines moderate-intensity physical activity as burning 3–5.9 METs [35]. Thus, a representative MET score for moderate physical activity was set at 5.0 in this study for young male non-athletic adults.

### 2.12. Statistical Analyses

Data are represented as box plots using SigmaPlot (version 14.5; Systat Software Inc., Palo Alto, CA, USA). Statistical differences were analyzed using the Mann–Whitney rank-sum test (SigmaPlot version 14.5). Statistical significance was set at *p* < 0.05. Because the data could not pass either the normality test (Shapiro–Wilk) or the equal variance test (Brown-Forsythe) in some parameter comparisons, non-parametric analysis was applied to all comparisons and correlation analyses. The data shown in the table are expressed as the mean ± SD. Statistical significance was not assessed because all pro-athletes achieved the maximum value.

Correlation between two indices was determined via Spearman’s correlation analysis, and correlation coefficients were calculated using the SigmaPlot software. Correlation strength was defined as follows: 0.8–1.0 = very strong, 0.6–0.79 = strong, 0.4–0.59 = moderate, 0.2–0.39 = weak, and 0–0.19 = very weak. Regression lines were fitted to scatter plots of TAC versus SMP or BMR in the control group using SigmaPlot.

Multi-comparison analysis was performed using the Kruskal–Wallis one-way ANOVA on ranks, followed by Dunn’s test as a post-hoc test for all pairwise comparisons.

## 3. Results

### 3.1. Basic Physical Specifications of Pro-Athletes

Age and body composition indices of the control and pro-athlete groups are shown in Figure 2. The control group was sex- and age-matched to the pro-athlete group. Their chronological age was first confirmed as the basis of this study. This comparison revealed that the control and pro-athlete groups were of the same generation (Figure 2a). No significant differences in the visceral fat level, body mass index, and BFP were observed between the groups (Figure 2b–d). However, SMP, bone mass weight, and BMR were significantly higher in the pro-athlete group than in the control group (Figure 2e–g). These statistical analyses between the two independent groups were conducted using the non-parametric Mann–Whitney rank-sum test.

The exercise volumes of the control and pro-athlete groups are shown in Table 1. Among pro-athletes who underwent intensive training and competed in soccer, the MET score was 120.0 per week. In non-athletic control individuals, MET scores were calculated 9.7 ± 13.7, which was approximately 8.1% of that of pro-athletes. A significant difference between the two groups (*p* < 0.001) was observed using the non-parametric Mann–Whitney rank-sum test.

Correlation coefficients between SMP or BFP and BMR in the control and pro-athlete groups are shown in Figure S1. Strong moderately negative correlations between SMP and BMR (Figure S1a,b) and strong moderately positive correlations between BFP and BMR (Figure S1c,d) were observed in the control and pro-athlete groups.

Blood cell counts and anemia indices in the control and pro-athlete groups are shown in Figure S2. ACD-A-treated blood samples were subjected to cell counting and HGB and HCT measurement. Platelet counts were significantly lower in the pro-athlete group than in the control group (Figure S2a). Additionally, RBC counts, HGB levels, and HCT values were significantly lower in the pro-athlete group than in the control group (Figure S2c–e). However, no significant differences in mean platelet volume (Figure S2b) and WBC counts (Figure S2f) were observed between the two groups.

Before TAC quantification, the influence of the preparation protocol was examined. The correlation between TAC quantification and the preparation protocol is shown in Figure S3. Strong and moderate positive correlations were observed between the TAC of ACD-A-treated PPP and PRP, or citrated PPP, samples. Thus, types of anticoagulants (ACD-A vs. citrate) did not influence the quantification of TAC.

TAC, ESR, and plasma protein, lactate, and glucose levels were also compared between the control and pro-athlete groups. Plasma indices and compositions related to inflammation and energy generation in the control and pro-athlete groups are presented in Figure S4. No significant differences were observed in TAC, ESR, and other plasma compositions between the two groups (Figure S4a–e). Only TAC values normalized by plasma protein levels showed a difference, being significantly lower in the pro-athlete group than in the control group (Figure S4f).

### 3.2. Correlations Between TAC and Body Composition Indices

**Figure 3:**
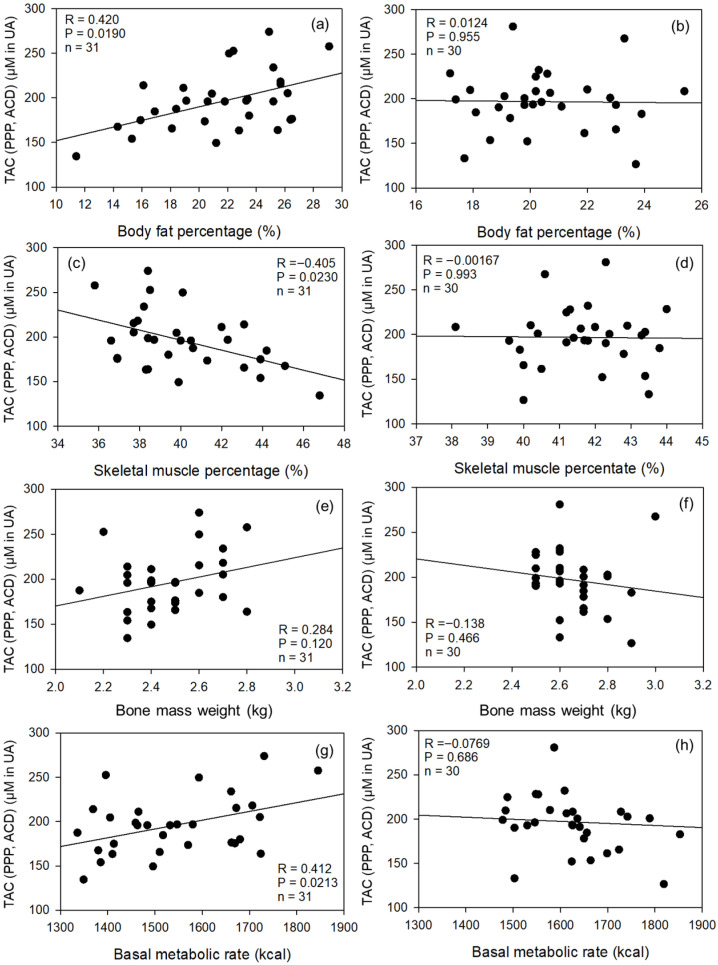
Correlation coefficients between TAC and body composition indices in the control and pro-athlete groups. (**a**,**b**) BFP vs. TAC in ACD-A-treated PPP samples. (**c**,**d**) SMP vs. TAC. (**e**,**f**) BMW vs. TAC. (**g**,**h**) BMR vs. TAC. Control (**a**,**c**,**e**,**g**) and pro-athlete (**b**,**d**,**f**,**h**) groups. *n* = 31 (controls) or 30 (pro-athletes).

Correlation coefficients between TAC and body composition indices in the control and pro-athlete groups are shown in Figure 3. Moderate correlations were observed only in the control group; TAC was positively correlated with BFP and BMR and negatively correlated with SMP in ACD-A-treated PPP samples. Very weak correlations were observed in the pro-athlete group.

Correlation coefficients between ESR values, an indicator of the inflammation levels, and blood cell counts in the control and pro-athlete groups are shown in Figure S5. Weak correlations (*p* > 0.05) were observed between ESR values and WBC counts in both groups (Figure S5a,b). Additionally, weak correlations were observed between ESR values and platelet counts only in the control group (Figure S5c), and very weak correlations were observed among all other combinations (Figure S5d–f).

### 3.3. Pro-Athletes’ Data Compensation Based on Non-Athletes’ Data

**Figure 4:**
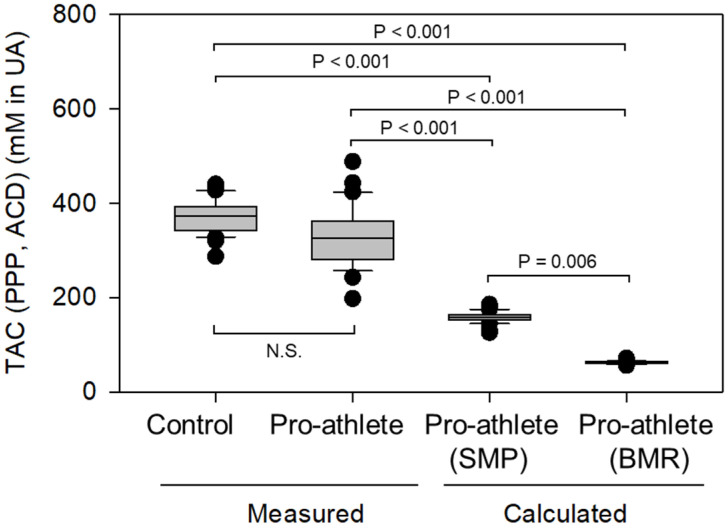
Measured TAC levels (left two boxes) and compensated TAC levels of pro-athletes based on the data of SMP and BMR (right two boxes). *n* = 31 or 30 for controls and pro-athletes.

Because different trends were observed in correlations between the control and pro-athlete groups, the measured values for pro-athletes were compensated using the correlation coefficients obtained in the control group. The measured and compensated TAC values were compared. In Figure 4, consistent with the data shown in Figure S4, no significant difference in TAC levels (raw data) was observed between the control and pro-athlete groups. However, given the considerable differences in body composition, particularly those closely associated with skeletal muscle, we propose adjusting raw TAC values to improve the interpretation of TAC data in pro-athletes. Each pro-athlete’s SMP or BMR value was substituted into the formulae of regression lines below to yield a compensated TAC value.

SMP: Compensated TAC = −0.385 × (SMP) + 233.645

BMR: Compensated TAC = 1.714 × (BMR) + 1204.286

This approach revealed that compensated TAC levels, regardless of BCI types (SMP or BMR), are substantially lower than the measured TAC levels (*p* < 0.001). These statistical differences were obtained using the Kruskal–Wallis one-way ANOVA on ranks, followed by Dunn’s test as a post-hoc test for all pairwise comparisons.

## 4. Discussion

In this study, we hypothesized that pro-athletes’ TAC levels would be higher than those of non-athletes. However, when comparing raw data on measured TAC levels, we found no significant difference. Thus, focusing on differences in body composition, we adjusted the raw TAC data using linear regressions from non-athletes relating SMP or BMR to TAC levels. Interestingly, the compensated TAC values were significantly lower than the raw TAC values, suggesting that pro-athletes may elevate their TAC above theoretical levels.

Consistent with previous reports [12,13,14,15], this study demonstrated enhanced physical characteristics in pro-athletes, including well-developed skeletal muscles and a high BMR, attributable to regular, intense exercise, although decreased RBC and platelet counts, HGB levels, and HCT values indicated mild anemia-like conditions. Thus, we raised a working hypothesis that TAC levels increase in response to continuous exercise-induced inflammatory stimuli in athletes. However, no significant differences were detected in non-normalized TAC or ESR levels. Although plasma protein levels were not significantly different between the two groups, TAC values normalized for plasma protein levels were significantly lower in the pro-athlete group than in the control group. These data are sufficiently interesting to prompt reconsideration of the interpretation of raw TAC data, because protein intake is crucial for anabolism, for example, building skeletal muscle by providing amino acids for synthesis, especially after exercise [36], as in the pro-athletes who participated in this study.

Regarding correlations with BCI, moderate correlations were observed between TAC and BFP, SMP, and BMR in the control group. In contrast, only weak or very weak correlations were observed in the pro-athlete group. These findings suggest that TAC levels are generally influenced by body composition, specifically skeletal muscle, in the majority of young adult males. In this sense, unidentified factor(s) may perturb this principle in pro-athletes. Therefore, raw TAC data were corrected using correlations observed in the control group to restore the “original” TAC value, which should be free of pro-athlete-specific factors. As observed in the TAC data normalized using plasma protein levels, calculated TAC levels in pro-athletes were substantially lower than those in controls.

Given this cross-sectional study design, discussing discrepancies between pro-athletes and non-athletes is challenging and subject to interpretive limitations, particularly regarding mechanisms. However, prudent consideration of the possible mechanism will provide hints for subsequent studies. We believe that ROS production should be considered a promising factor. In particular, the balance between TAC and ROS may be critical, because TAC was not significantly lower in the pro-athlete group than in the control group. Contracting skeletal muscles generate ROS, which trigger mitochondrial biogenesis-regulating signaling pathways and the expression of numerous genes encoding mitochondrial proteins and antioxidant enzymes [37]. High ROS levels are associated with skeletal muscle damage and impaired muscle function [38]. However, to protect against ROS-induced muscle damage, the body has compensatory mechanisms that enhance adaptive responses by upregulating enzymes such as superoxide dismutase, catalase, and glutathione peroxidase, which neutralize free radicals and increase antioxidant capacity during regular exercise [27]. Regular exercise stimulates the generation of new mitochondria that efficiently produce energy and ROS. ROS do not always hinder tissue regeneration; they may also support it, depending on the balance between ROS and antioxidant enzyme levels that affect TAC. Similar phenomena have been reported in untrained humans [39]. Furthermore, a systematic review and meta-analysis revealed that, regardless of exercise intensity, volume, type, or population studied, antioxidant indicators tended to increase and prooxidant indicators tended to decrease after training, suggesting that exercise training induces an antioxidant effect [40]. Further molecular investigations, preferably employing a longitudinal study design, may help clarify whether regular exercise or body composition is more influential in athletes’ TAC regulation when assessing the suitability of athletes for PRP regenerative therapy.

Regarding physical activity, the difference between non-athletic and pro-athletic adults lies in the latter’s greater engagement in intense exercise. In muscles, ROS production generally depends on muscle volume [41]. To further develop their sports performance, athletes engage in regular and intense exercise. Along with increases in muscle mass, mitochondrial number (per muscle cell) and subsequent ROS production increase, thereby inducing low-grade inflammation [42] and muscle damage. Interestingly, regular exercise also increases TAC levels to scavenge ROS and minimize muscle damage [27,29]. In this scenario, pro-athletes were expected to have higher TAC than non-athletic controls.

In contrast, the non-normalized TAC levels in pro-athletes did not differ from those of the controls, whereas TAC levels normalized using plasma protein levels were lower in pro-athletes than in controls. Further in-depth investigations with larger sample sizes are required to address these unexpected results. However, based on correlations with BCI, pro-athletes’ “basic” TAC levels could be much lower than the measured values. This suggests that regular intense exercise may elevate TAC levels along with muscle development. In addition, TAC levels cannot be directly compared across “counterparts” with different body compositions.

To translate this finding into practical applications, further investigations using a large sample size across athletic levels, from recreational to elite, are needed. In previous studies, we proposed, based on analyses of pro-athletes, that polyphosphates and anti-inflammatory cytokines could serve as distinct markers of successful PRP therapy. SMP- (or BMR)-compensated TAC, in combination with these and other markers, may help identify non-responders to PRP therapy in the future, although this is not currently applicable.

### Limitations

This study has several limitations. First, the sample size was relatively small to reach definitive conclusions, owing to the difficulty in enrolling a sufficient number of professional soccer athletes beyond specific team boundaries without the support of commissioners and individual team headquarters. Therefore, our method is currently the most reasonable and practical for collecting data from athletic teams.

Second, immediate point-of-care testing using fresh samples is favorable and often mandated for laboratory testing of blood samples. For several practical reasons, sample handling was delayed for approximately 20 h. Thus, the possible influence of this delay on several test datasets cannot be excluded.

Third, TAC levels are generally most influenced by the quality and quantity of diets [33]. Lifestyle, supplement use, and training schedules could be significant factors, although we found no participants taking supplements. Thus, because their lifestyle and other factors could not be strictly controlled, confounding may be present in our analysis.

Additionally, it should be noted that only male participants were included in the study.

## 5. Conclusions

For practical reasons, the sample size was too small to draw a definitive conclusion. However, given that plasma TAC is generally associated with muscle-associated body composition (SMP or BMR) in controls, the current data suggest that pro-athletes may have elevated TAC levels when the control group’s scales are applied.

## Figures and Tables

**Table 1:** Exercise volume (physical activity) of non-athletic control and pro-athletes.

Group	METs (Per Week)
Control	9.7 ± 13.7 (*n* = 31)
Pro-athlete	120.0 ± 0.0 (*n* = 30)

## Data Availability

Data is contained within the article or Supplementary Materials.

## Supplementary Materials

The following supporting information can be downloaded at: https://www.mdpi.com/article/10.3390/sports14020045/s1, Figure S1: Correlation coefficients between SMP or BFP and basal metabolic rate (BMR) in the control and pro-athlete groups; Figure S2: Blood cell counts and anemia indices in the control and pro-athlete groups. Blood samples were collected in the presence of ACD-A as an anticoagulant; Figure S3: Correlation coefficient between the total antioxidant capacity (TAC) and preparation protocol; Figure S4: Plasma indices and compositions related to inflammation and energy generation in the control and pro-athlete groups; Figure S5: Correlation coefficients between ESR values and blood cell counts in the control and pro-athlete groups.

## Abbreviations

The following abbreviations are used in this manuscript:

PRP	platelet-rich plasma
PPP	platelet-poor plasma
pro-athlete	professional athlete
RBC	red blood cell
WBC	white blood cell
PLT	platelet
VFL	visceral fat levels
BCI	body composition index
BMI	body mass index
BFP	body fat percentage
SMP	skeletal muscle percentage
BMW	bone mass weight
BMR	basal metabolic rate
WB	whole blood
TAC	total antioxidant capacity
ESR	erythrocyte sedimentation rate
UA	uric acid
PBS	phosphate-buffered saline
VTE	venous thromboembolism
HBG	hemoglobin
HCT	hematocrit
MPV	mean platelet volume
MET	metabolic equivalent of task
