# Supplementary material for: Is Plasma Total Antioxidant Capacity Elevated in Professional Soccer Athletes?: A Cross-Sectional Study
Source: Sports (Basel). 2026 Jan 23;14(2):45. doi: 10.3390/sports14020045 (PMC12945066; doi:10.3390/sports14020045)
Supplement: Supplementary file 1 [file sports-14-00045-s001.zip › sports-4083485-supplementary.pdf]

Supplemental data

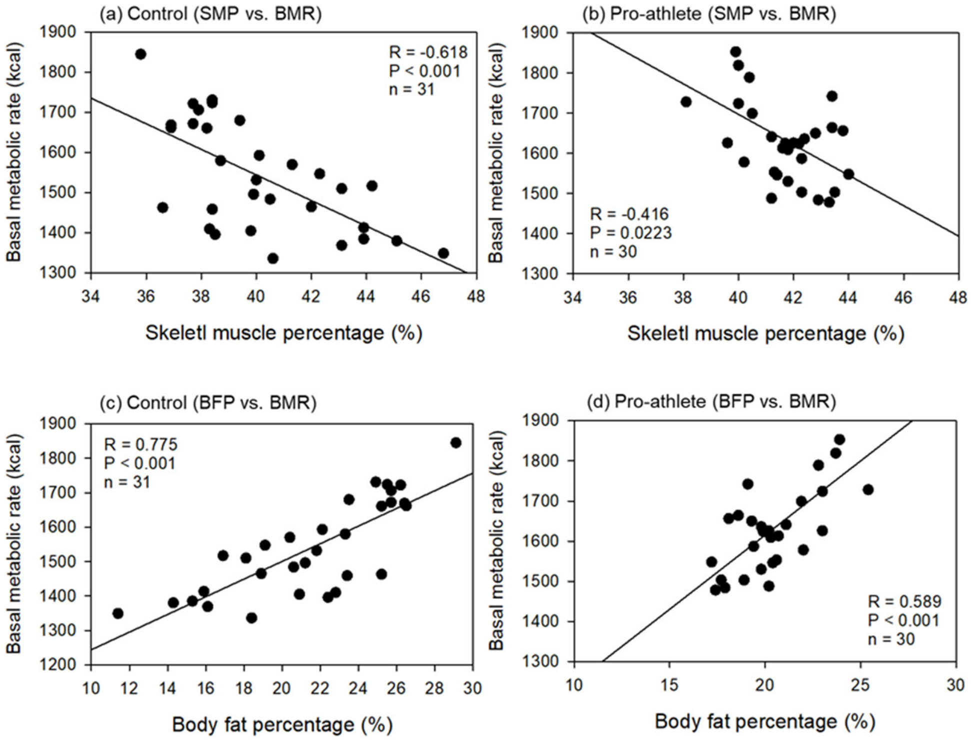

Figure S1. Correlation coefficients between SMP or BFP and basal metabolic rate (BMR) in the control and pro-athlete groups. (a, b) SMP vs. BMR in the control and pro-athlete groups. (c, d) BFP vs. BMR in the control and pro-athlete groups. Control (a, c) and pro-athlete (b, d) groups. Correlation coefficients and P values were determined using the non-parametric Spearman's correlation coefficient. n = 31 (controls) or 30 (pro-athletes).

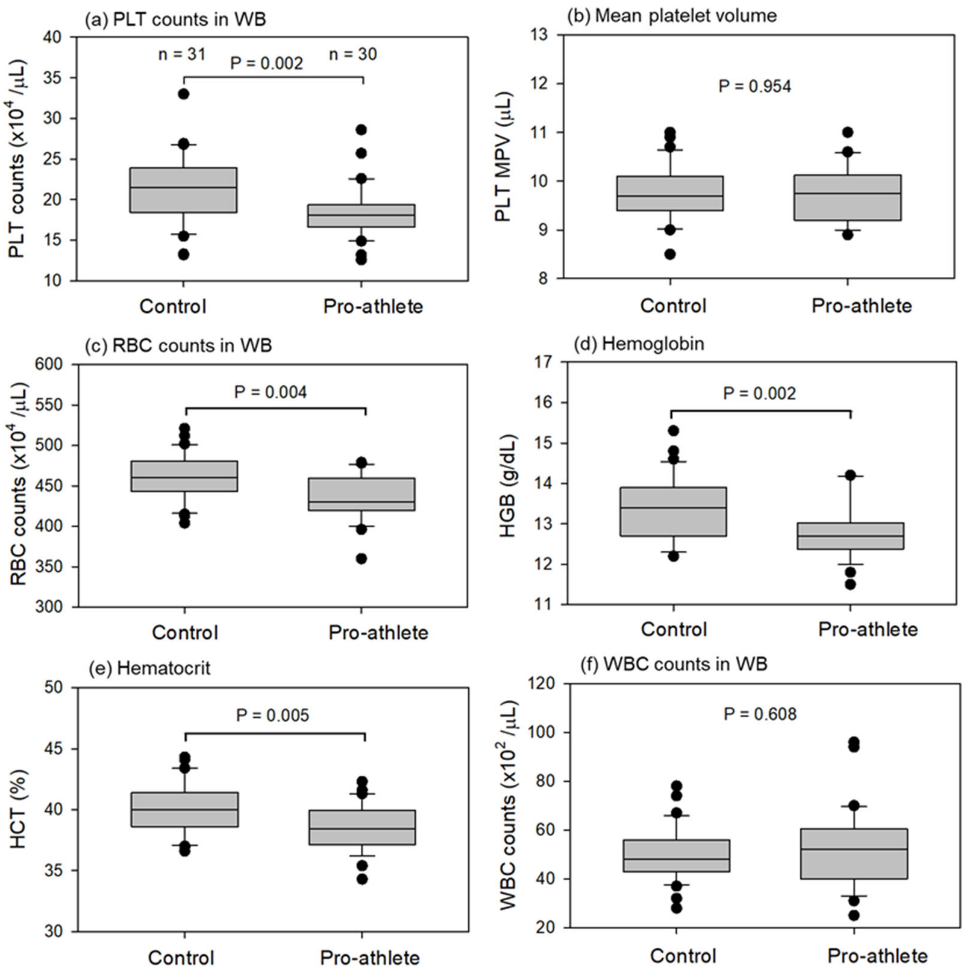

Figure S2. Blood cell counts and anemia indices in the control and pro-athlete groups. Blood samples were collected in the presence of ACD-A as an anticoagulant. (a) Platelet (PLT) counts in whole blood (WB) samples. (b) Mean platelet volume (MPV). (c) Red blood cell (RBC) counts in WB samples. (d) Hemoglobin (HGB) levels. (e) Hematocrit (HCT) values. (f) White blood cell (WBC) counts in WB samples. n = 31 (controls) or 30 (pro-athletes).

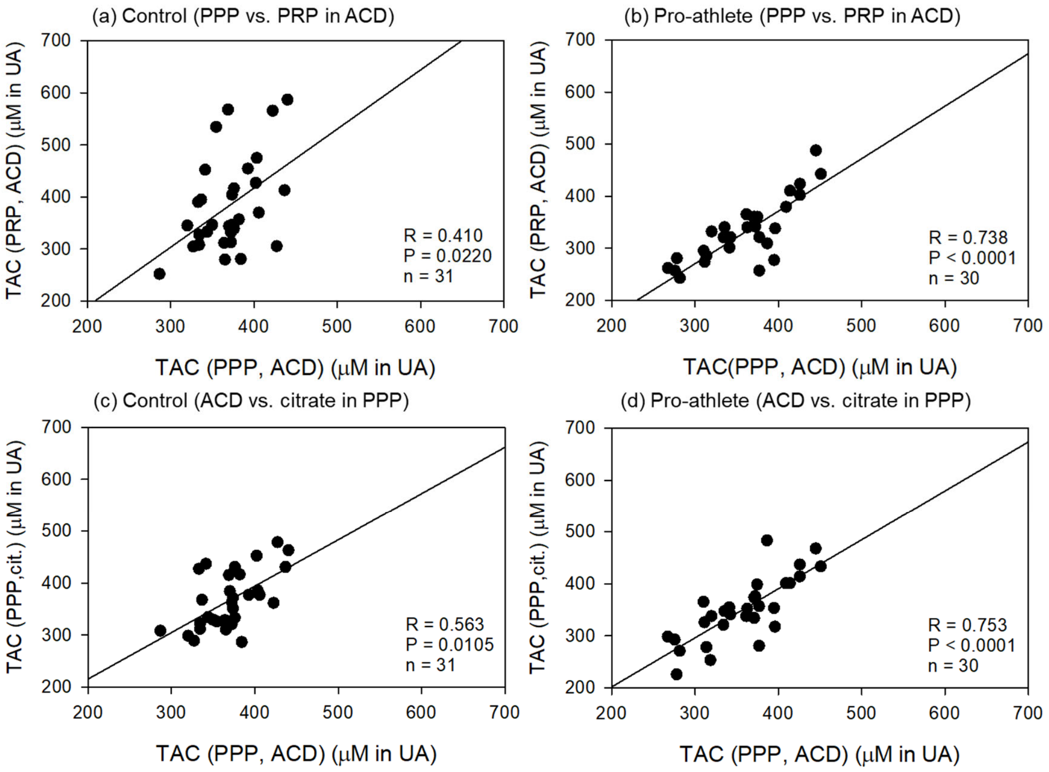

Figure S3. Correlation coefficient between the total antioxidant capacity (TAC) and preparation protocol. Platelet-poor plasma (PPP) and platelet-rich plasma (PRP) samples were prepared from the blood samples collected using ACD-A or citrate (cit.) as anticoagulants. (a, b) PPP vs. PRP in the ACD-A-treated samples of the control and pro-athlete groups. (c, d) ACD-A vs. citrate in the PPP samples of the control and pro-athlete groups. Control (a, c) and pro-athlete (b, d) groups. n = 31 (controls) or 30 (pro-athletes).

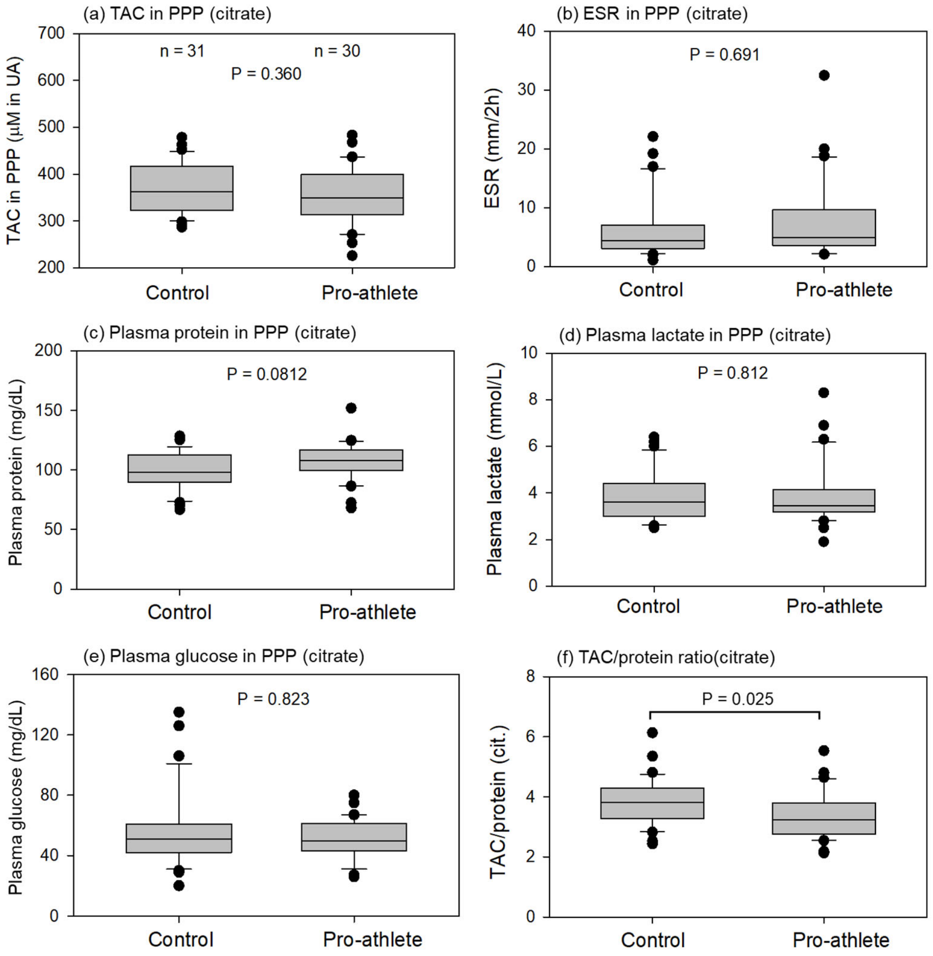

Figure S4. Plasma indices and compositions related to inflammation and energy generation in the control and pro-athlete groups. (a, b) TAC and erythrocyte sedimentation rate (ESR) in PPP samples prepared from citrated blood samples. (c, d, e) Plasma protein, lactate, and glucose levels in PPP samples prepared from citrated blood samples. (f) TAC values normalized by plasma protein levels. Statistical analyses were conducted using the non-parametric Mann–Whitney U test. n = 31 (controls) or 30 (pro-athletes).

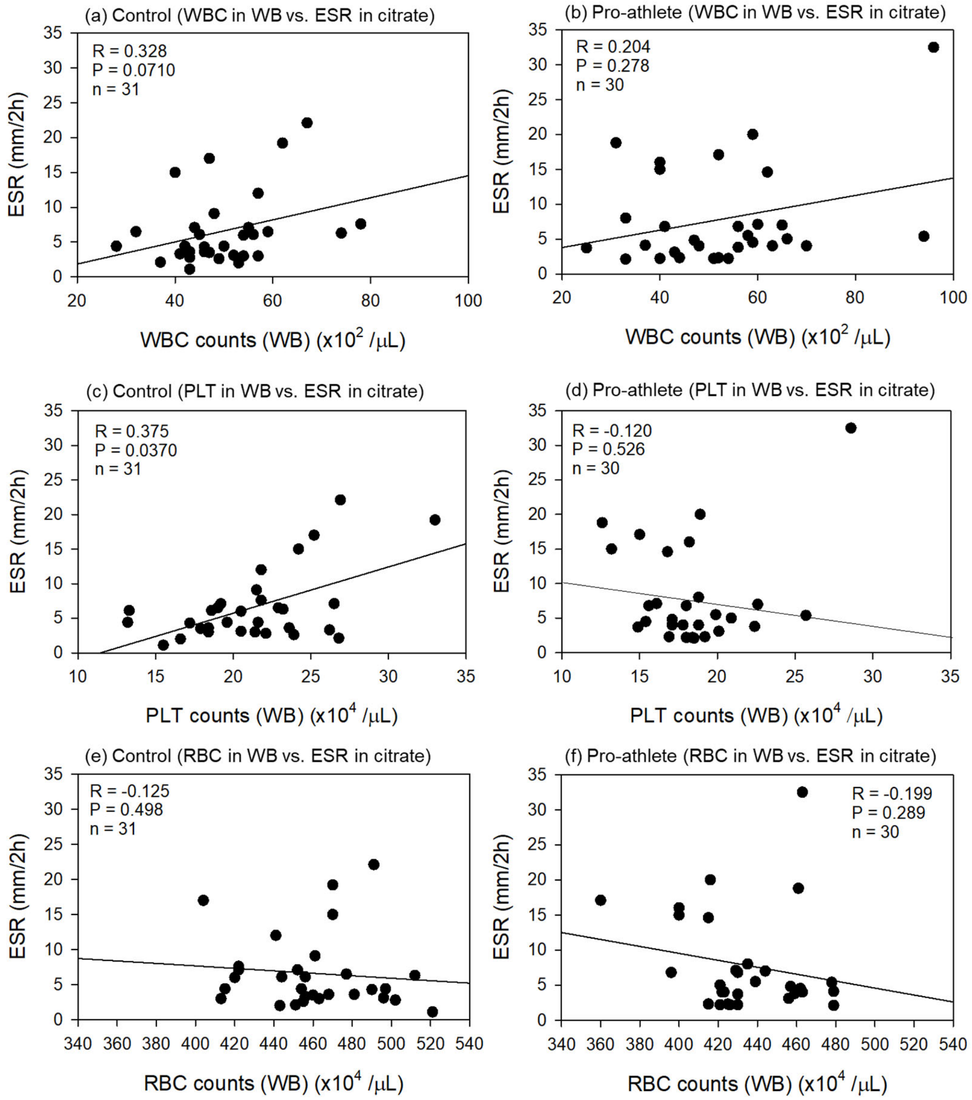

Figure S5. Correlation coefficients between ESR values and blood cell counts in the control and pro-athlete groups. (a, b) WBC counts in whole blood samples vs. ESR values in citrate-prepared PPP samples. (c, d) PLT counts vs. ESR values. (e, f) RBC counts vs. ESR values. Control (a, c, e) and pro-athlete (b, d, f) groups. n = 31 (controls) or 30 (pro-athletes).
